# First molecular confirmation of *Spirometra mansoni* (Cestoda: Diphyllobothriidea) in Indonesian cobras (*Naja sputatrix* and *Naja sumatrana*)

**DOI:** 10.1186/s13071-026-07506-x

**Published:** 2026-07-09

**Authors:** Ryanka Edila, Lucia Tri Suwanti, Caroline Jepkorir Kibet, Nnabuife Bernard Agumah, Mustofa Helmi Effendi, Seongjun Choe

**Affiliations:** 1 Doctoral Program of Veterinary Science, Faculty of Veterinary Medicine, Universitas Airlangga, Jl. Mulyorejo, Kampus C, Surabaya, 60115 Indonesia; 2 Division of Veterinary Parasitology, Faculty of Veterinary Medicine, Universitas Airlangga, Jl. Mulyorejo, Kampus C, Surabaya, 60115 Indonesia; 3 Institute of Tropical Diseases, Universitas Airlangga, Jl. Mulyorejo, Kampus C, Surabaya, 60115 Indonesia; 4 Ichthyology Section, Zoology Department, National Museums of Kenya, P.O. Box 40658–00100, Nairobi, Kenya; 5 Department of Medical Laboratory Science, Faculty of Health Science and Technology, Ebonyi State University, Abakaliki, Nigeria; 6 Division of Veterinary Public Health, Faculty of Veterinary Medicine, Universitas Airlangga, Jl. Mulyorejo, Kampus C, Surabaya, 60115 Indonesia; 7 Department of Parasitology, School of Medicine, Chungbuk National University, Cheongju, Chungcheongbuk-do 28644 Republic of Korea

**Keywords:** *Spirometra mansoni*, *Naja sputatrix*, *Naja sumatrana*, Cestoda, Zoonoses, Indonesia, Phylogenetic analysis, Wildlife trade

## Abstract

**Background:**

Cobras (*Naja* spp.) are widely consumed and traded in Southeast Asia, particularly in Indonesia, yet their role in the transmission of zoonotic cestodes of the genus *Spirometra* remains unresolved at the species level. Accurate molecular identification is essential to clarify host associations, population connectivity, and potential public health implications.

**Methods:**

Plerocercoids were collected from *Naja sputatrix* (East Java) and *Naja sumatrana* (West Sumatra). Specimens were characterised morphologically and subjected to mitochondrial cytochrome c oxidase subunit I (cox1) sequencing. Phylogenetic analyses at the species level were conducted to determine the identity of the isolated cestodes and assess genetic relationships among isolates. Pairwise genetic distances were calculated to examine whether genetic differentiation correlated with host species.

**Results:**

All newly generated sequences were consistently placed within the *Spirometra mansoni* clade with strong nodal support. Despite observable differences in larval body size between host species, all 15 isolates were distributed across only two *cox1* haplotypes (*P*-distance = 0.000 within haplotypes, 0.020 between haplotypes), both of which were shared between *N. sputatrix* and *N. sumatrana*, indicating no host-associated genetic differentiation between the two examined cobra species. Indonesian isolates exhibited high genetic similarity to previously reported *S. mansoni* sequences from other domestic and wild vertebrates (*P*-distance ≤ 0.026), as well as from geographically distant regions including Australia, China, and South America. These findings provide the first molecular confirmation of *S. mansoni* in *N. sputatrix* and the first record of *Spirometra* infection in *N. sumatrana*.

**Conclusions:**

*Spirometra mansoni* was confirmed to infect two cobra species in Indonesia (*N. sputatrix* and *N. sumatrana*) without evidence of host-specific genetic partitioning. The limited genetic differentiation across regions suggests substantial population connectivity, potentially facilitated by anthropogenic activities such as wildlife exploitation and trade. Cobras may represent an epidemiologically relevant interface linking sylvatic transmission cycles with human exposure. Molecular surveillance of *Spirometra* in wildlife entering food chains is warranted to better assess zoonotic risk.

**Graphical Abstract:**

**Figure:**
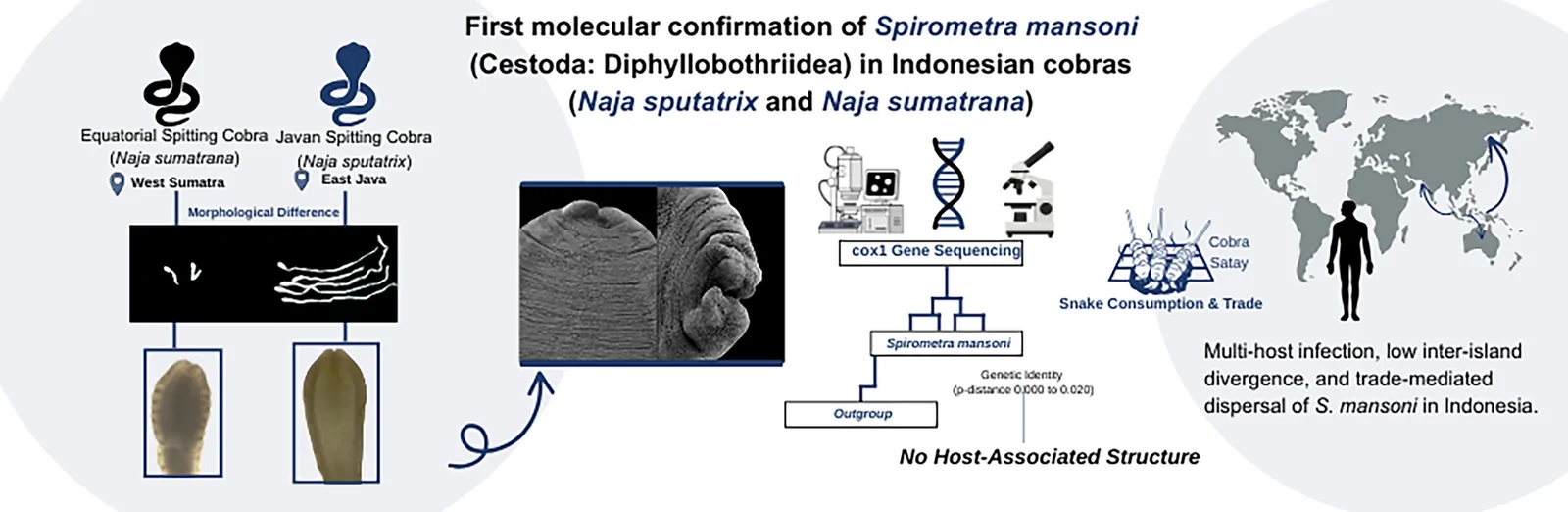

**Supplementary Information:**

The online version contains supplementary material available at https://doi.org/10.1186/s13071-026-07506-x.

## Background

The consumption of snakes has long been embedded in the cultural, subsistence, and medicinal practices of many Southeast and East Asian societies and continues to persist in modern times [1, 2]. Among the reptiles most frequently utilised for food and traditional medicine are cobras of the genus *Naja* Laurenti, 1768, commonly referred to as “true cobras” [3, 4]. Indonesia represents a particularly important centre for the exploitation and trade of venomous snakes. It is currently recognised as the world’s leading exporter of both venomous snakes and snakes overall, with the Javan spitting cobra, *Naja sputatrix* Boie, 1827, being among the most heavily utilised species, with export quantities estimated at approximately 2.2 million individuals [5]. This species is native to Java, Bali, and the Lesser Sunda Islands [6] and is widely traded for meat, including its use in the so-called “cobra satay” industry operating in various regions, including several locations within the Special Capital Region of Jakarta [7]. Indonesian-sourced cobra meat is also exported and used in snake-based food industries in other Asian regions [3].

In addition to *N. sputatrix*, the equatorial spitting cobra, *Naja sumatrana* Müller, 1887, is also commonly consumed in Indonesia, particularly for perceived medicinal benefits associated with traditional beliefs [2]. *N. sumatrana* is distributed across the Malay Peninsula, Sumatra, southern Thailand, and parts of Borneo. Both species were formerly included within a broader *Naja naja*–*sputatrix* species complex, but subsequent taxonomic revisions have recognised *N. sumatrana* as a distinct species. Under the revised taxonomy, cobra populations in Indonesia are assigned to *N. sputatrix*, whereas those in Thailand and Indochina are assigned to *Naja siamensis* Peters, 1861, with *N. sumatrana* recognised as a separate lineage distributed across the Malay Peninsula, Sumatra, and adjacent regions [4].

Snakes are among the most frequently reported second intermediate or paratenic hosts of zoonotic tapeworms of the genus *Spirometra* Faust, Campbell et Kellogg, 1929, parasites of major medical and veterinary significance worldwide [8]. Adult *Spirometra* inhabit the small intestines of carnivores, whereas their larval stages (spargana) occur in a wide range of vertebrate hosts, causing the disease known as sparganosis [9, 10]. The life cycle is complex and involves freshwater copepods (e.g. Cyclopidae) as first intermediate hosts, various vertebrates (amphibians, reptiles, or mammals) as second intermediate or paratenic hosts, and carnivorous mammals as definitive hosts [11, 12]. Sparganosis is a disease of humans and animals that can result in severe clinical manifestations, including subcutaneous, ocular, and cerebral forms, which may cause long-term morbidity or life-threatening complications [11, 13]. More than 2,500 human cases have been reported worldwide [14], most of them linked to dietary and cultural practices involving the consumption of infected animals [15–17].

In Indonesia, *Spirometra* infections in snakes have been frequently reported, often at remarkably high prevalence rates (ranging from 56.7 to 82.3% in *N. sputatrix*). Surveys of wild-caught snakes from multiple regions have documented widespread infections, although identifications were generally limited to the genus level [18–20]. In *N. sputatrix*, high prevalence of *Spirometra* plerocercoids has been reported repeatedly, reaching 56.7% in Banyuwangi, East Java [21], and 60% in subsequent surveys without species-level confirmation [22]. Similarly, an epidemiological helminth survey of *N. sputatrix* in Sidoarjo, East Java, detected *Spirometra* larvae in 82.3% of examined snakes [8].

Despite these high infection rates, the prevalence data cited above were based on morphological diagnosis at the genus level, which is achievable through recognition of general larval characteristics including a bothriate cephalic region and the absence of hooks. However, species-level identification within *Spirometra* is notoriously difficult based on morphology alone, owing to the overall morphological conservatism of spargana across species. Accurate species-level identification therefore relies primarily on molecular approaches**.** Moreover, geographical distribution and host range of *Spirometra* species remain poorly understood [12]. To date, molecular data on *Spirometra* species infecting snakes in Indonesia remain extremely limited. Only two molecular reports are currently available. The first involved domestic dogs from Samosir, North Sumatra, in which isolates were misidentified as *Spirometra erinaceieuropaei* [23]. More recently, the first prospective molecular confirmation of *S. mansoni* in the country was provided, with the parasite detected in 84% of examined Javanese keelback water snakes (*Fowlea melanzosta* Gravenhorst, 1807) [24]. In contrast, no molecularly confirmed records of *Spirometra* from *N. sputatrix* are currently available, and infections in *N. sumatrana* have not been reported at any taxonomic level.

Given the high levels of cobra consumption, extensive international trade in snake meat, and the severe clinical consequences of human sparganosis, the lack of species-level molecular data from key snake hosts represents a critical knowledge gap. The present study addresses this gap by providing molecular identification of *Spirometra* from *N. sputatrix* and reporting, for the first time, *Spirometra* infection in *N. sumatrana* based on *cox*1 mitochondrial DNA data. These findings contribute essential baseline information for understanding transmission pathways, assessing zoonotic risk, and improving surveillance of foodborne parasitic infections associated with snake consumption in Indonesia and beyond.

## Methods

### Parasite material

**Fig. 1:**
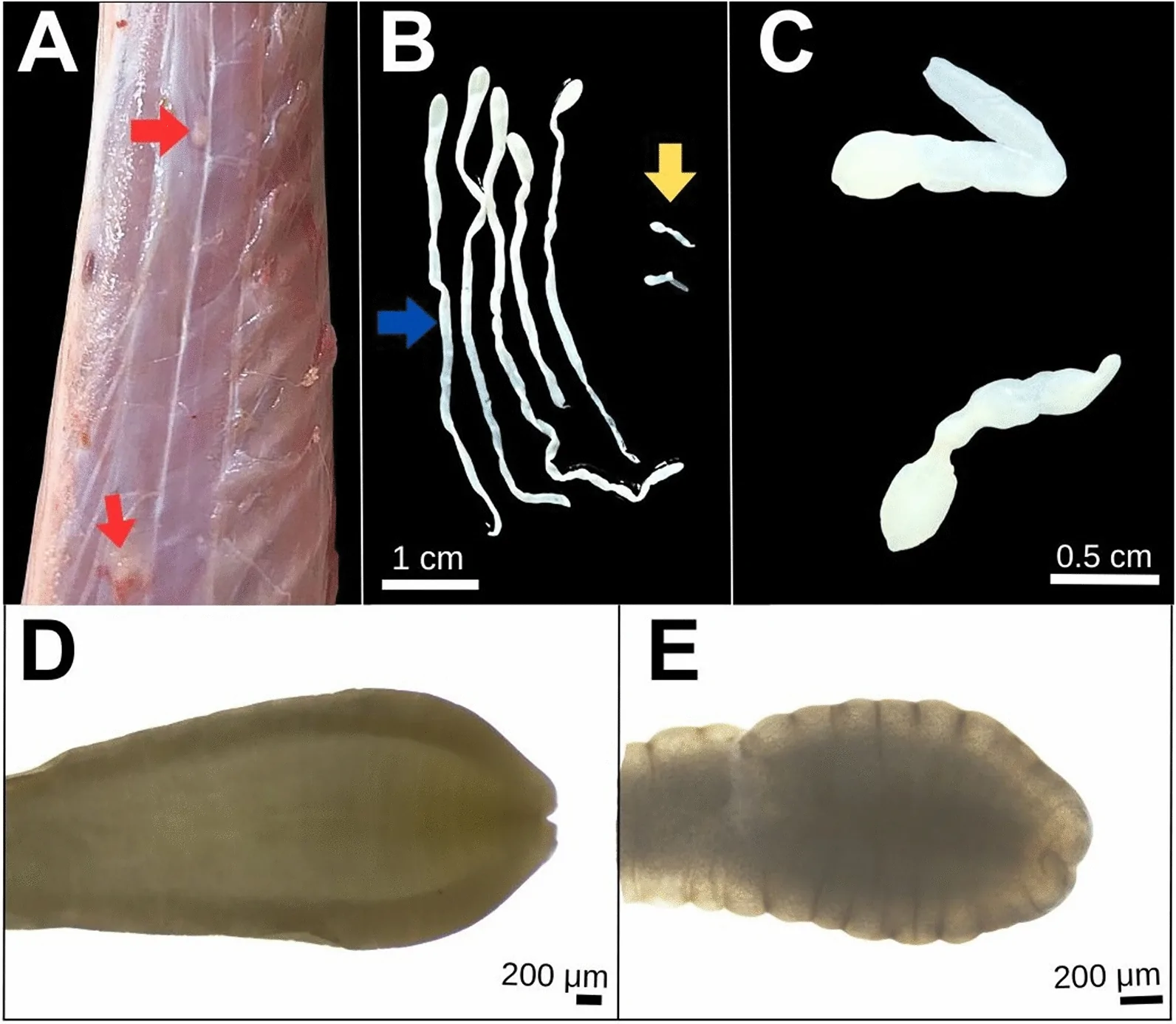
*Spirometra mansoni* plerocercoids recovered from *Naja sumatrana* and *Naja sputatrix* in Indonesia. **A** Plerocercoids embedded in the musculature of *N. sumatrana* (arrows). **B** Size comparison of plerocercoids from the two host species (blue arrows, *N. sputatrix*; yellow arrows, *N. sumatrana*). **C** Isolated plerocercoids from *N. sumatrana*. **D**, **E** Anterior region of plerocercoids under light microscopy: **D** isolate from *N. sputatrix*; **E** isolate from *N. sumatrana*. Scale bars as indicated

Plerocercoid specimens analysed in this study originated from two species of cobras, *N. sputatrix* and *N. sumatrana*. A total of 12 plerocercoid specimens from *N. sputatrix* were obtained from archived material previously collected in Sidoarjo, East Java, Indonesia, and reported in an earlier epidemiological study [8]. These specimens had been preserved in 80% ethanol and were re-examined exclusively for molecular analyses in the present study. An additional five plerocercoid specimens from *N. sputatrix*, preserved in 10% formalin, were obtained from the same archived collection and used exclusively for scanning electron microscopy. In addition, three plerocercoid specimens were recovered from the musculature of *N. sumatrana* (Fig. 1A) obtained opportunistically after being found dead along the Batang Mangoi River, West Sumatra, Indonesia (− 0.6027° S, 100.1889° E). Species identity was determined based on external morphological characters, including scalation, colouration, and hood morphology, following Wüster [25] and was further corroborated by the collection locality in West Sumatra, which falls within the confirmed distributional range of this species [4]. Necropsy was performed immediately in the field to minimise post-mortem degradation. The recovered plerocercoids were gently rinsed in physiological saline to remove debris and subsequently preserved in 80% ethanol until molecular processing. No animals were killed or euthanised for the purposes of this study.

### Morphological examination

Plerocercoid morphology was documented using light microscopy (LM) and scanning electron microscopy (SEM). Prior to further analyses, overall body dimensions (body length and body width) were recorded using a digital calliper. For LM, specimens from both host species were mounted in glycerol jelly and examined under a CX-23 light microscope (Olympus, Tokyo, Japan), focusing on general body form, cephalic region morphology, and the junction between the cephalic region and neck. SEM was performed exclusively on formalin-fixed specimens from *N. sputatrix* to characterise tegumental surface architecture, including the morphology and distribution of spinitrices, which were assessed qualitatively and compared with published descriptions of *Spirometra* spargana [26]. Specimens from *N. sumatrana* were not processed for SEM owing to larval fragility and limited material availability, all of which was allocated to molecular analyses. For SEM preparation, specimens were rinsed three times in phosphate-buffered saline (PBS), fixed in 2.5% glutaraldehyde, and subsequently washed in PBS. Dehydration was carried out through a graded ethanol series (70–100%), followed by immersion in isoamyl acetate. Samples were then air-dried overnight in a sealed container containing silica gel, sputter-coated with gold using a Quorum Q150T ES Plus coater (Quorum Technologies Ltd., UK), and examined using a GeminiSEM 560 scanning electron microscope (Carl Zeiss, Germany).

### Molecular procedures

Genomic DNA was extracted from a total of 15 plerocercoids, using midbody tissue sections to minimise contamination and maximise DNA quality. DNA extraction was performed with the Clear-S^™^ Quick-DNA Extraction Kit (InVirusTech, Gwangju, South Korea) following the manufacturer’s instructions, with two modifications previously applied to archival helminth tissue [8] to maximise DNA yield from limited biological material: (1) glycogen was added at 1 μL instead of the recommended 0.5 μL to facilitate nucleic acid co-precipitation, and (2) the final elution volume was reduced to 20 μL to increase DNA concentration. DNA concentration and purity were assessed using a NanoDrop 2000 spectrophotometer (Thermo Fisher Scientific, USA).

A partial fragment of the mitochondrial cytochrome c oxidase subunit I (*cox1*) gene was amplified using primers p1f (5′-TGGTTTTTTGGACATCCTGAA-3′) and p1r (5′-ATCACATAATGAAAGTGAGCC-3′), targeting an approximately 440 bp region corresponding to positions 707–1146 of the *cox1* gene [27]. Repeated amplification of longer *cox1* fragments yielded no consistent product across samples; amplification was therefore restricted to this 440 bp fragment, which produced reliable results across all 15 specimens**.**

PCR reactions were carried out in 30 μL volumes containing 6 μL of EzPCR^™^ XO 5 × PCR Master Mix (ELPIS-BIOTECH®, Daejeon, South Korea), 2 μL of each primer, 2 μL of genomic DNA (> 20 ng μL⁻^1^), and 18 μL of nuclease-free water. Thermal cycling conditions consisted of an initial denaturation at 94 °C for 3 min, followed by 35 cycles of denaturation at 94 °C for 40 s, annealing at 46 °C for 40 s, and extension at 72 °C for 1 min, with a final extension step at 72 °C for 4 min, following Jeon et al. [27]. PCR products were visualised on 1% agarose gels stained with ethidium bromide, and gel documentation was performed using a Gel Doc^™^ XR + system (Bio-Rad, Hercules, CA, USA).

### Genetic analyses

PCR amplicons were purified and Sanger-sequenced using a commercial sequencing service (Cosmogenetech, Seoul, South Korea). Forward and reverse reads were visually inspected and assembled into consensus sequences using Geneious Prime^®^ 2025.0.2 [28]. All sequences were deposited in GenBank (accession numbers PX458870–PX458874 and PX859754–PX859760 for *N. sputatrix* isolates, and PX859761–PX859763 for *N. sumatrana* isolates; Additional file 2: Table S2). Multiple sequence alignments were generated using MAFFT (plugin v1.5.0) [29] as implemented in Geneious Prime^®^ 2025.0.2.

Phylogenetic relationships among *Spirometra* species were inferred using both Bayesian inference (BI) and Maximum Likelihood (ML) approaches. The dataset comprised the 15 newly generated isolates together with representative published sequences of all currently accepted *Spirometra* species, selected based on alignment compatibility with the study sequences and their inclusion in recent authoritative phylogenetic syntheses of the genus [8, 9, 30]; full details are provided in Additional file 2: Table S2. The best-fitting substitution model (TN + F + I + G4) was selected under the Bayesian Information Criterion (BIC) using ModelFinder [31] as implemented in IQ-TREE v2.2.0. Bayesian inference was performed in MrBayes v3.2.6 [32] using four Markov chains run for 10,000,000 generations, with trees sampled every 1,000 generations and the first 25% of samples discarded as burn-in. ML inference was conducted in IQ-TREE v2.2.0 [33], with nodal support assessed using 1,000 ultrafast bootstrap replicates (UFBoot) [34]. *Dibothriocephalus nihonkaiensis* (GenBank: AB821272) served as the outgroup in both analyses. Trees were visualised in FigTree v1.4.4 [35].

Evolutionary divergence among isolates was estimated as uncorrected p-distances in MEGA 11 [36]. P-distances were calculated incorporating both transitions and transversions under a uniform rate among sites, with pairwise deletion applied to gaps and missing data across all three codon positions. Variance was not estimated, as pairwise distances were employed for descriptive quantification of divergence levels rather than formal hypothesis testing.

## Results

### Morphological observations

**Fig. 2:**
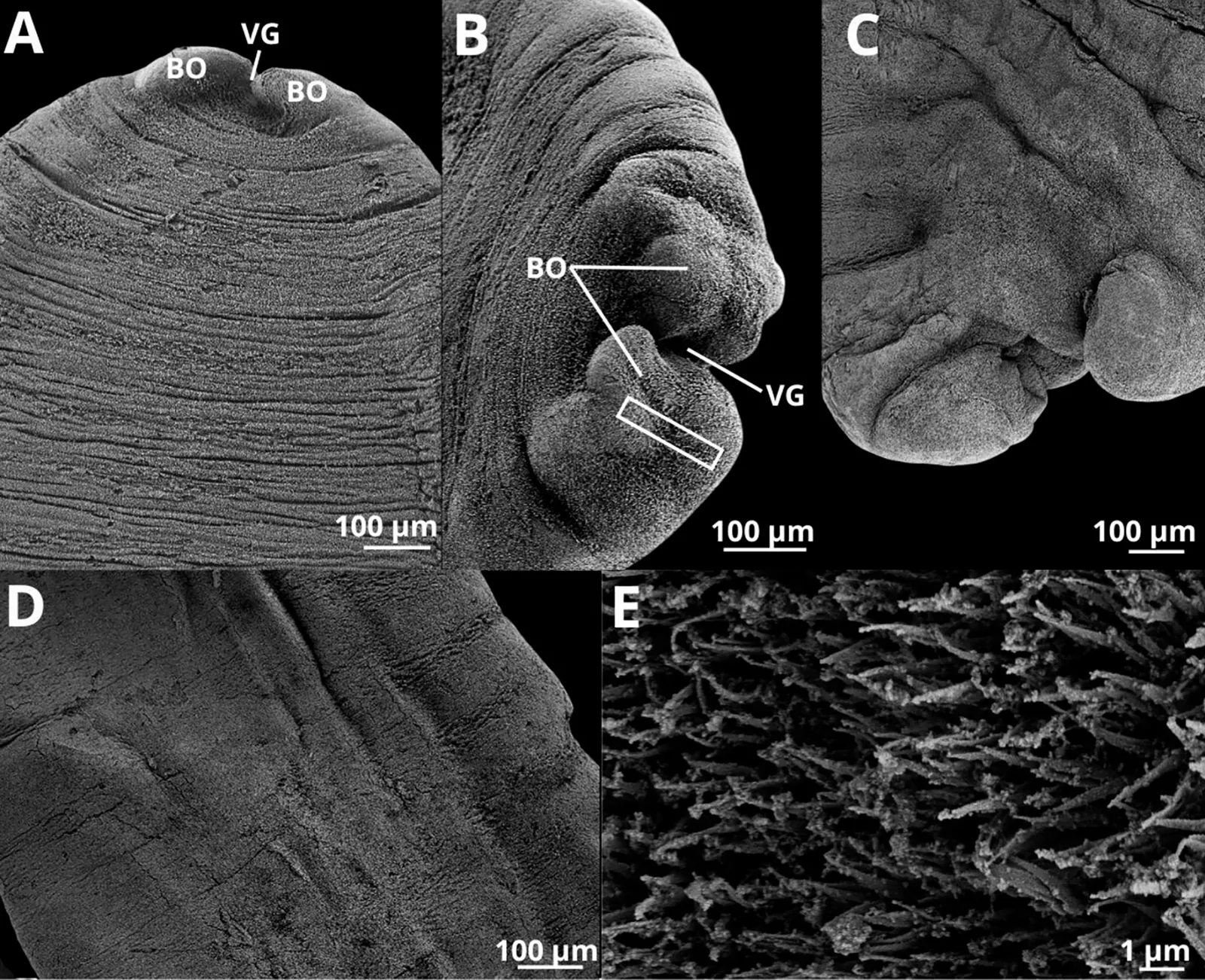
Scanning electron microscopy (SEM) of *Spirometra mansoni* plerocercoids from *Naja sputatrix*. **A** Anterior view of the cephalic region. **B** Lateral view of the cephalic region showing paired bothria and a rudimentary ventral groove. **C** Posterior region of the plerocercoid body. **D** External body surface with transverse wrinkling pattern. **E** Conical spinitrices densely distributed across the tegument (boxed area in **D**). *Bo* bothria, *VG* ventral groove. Scale bars as indicated

Spargana recovered from *N. sputatrix* and *N. sumatrana* were whitish in colour and differed markedly in body size (Fig. 1B, C). Spargana from *N. sputatrix* measured 40–75 mm in length and 1.8–2.2 mm in maximum width, whereas those from *N. sumatrana* were considerably smaller, measuring 9–13 mm in length and 1.2–1.41 mm in width. In both species, the cephalic region bore two distinct bothria, and the junction between the cephalic region and neck was slightly constricted (Fig. 1D, E). No additional qualitative differences in gross morphology were observed between larvae from the two host species. Scanning electron microscopy of spargana from *N. sputatrix* revealed well-developed bothria with a distinct median invagination (Fig. 2A, B). The posterior end of the body exhibited a shallow terminal indentation (Fig. 2C). The tegumental surface was characterised by prominent transverse and irregular wrinkles (Fig. 2D). Conical spinitrices, with an exposed length of approximately 2 μm as estimated from SEM micrographs using the calibrated scale bar (Fig. 2E), consistent with published descriptions [26], were strumose and densely distributed over the entire tegumental surface.

### Molecular phylogenetic analysis

**Fig. 3:**
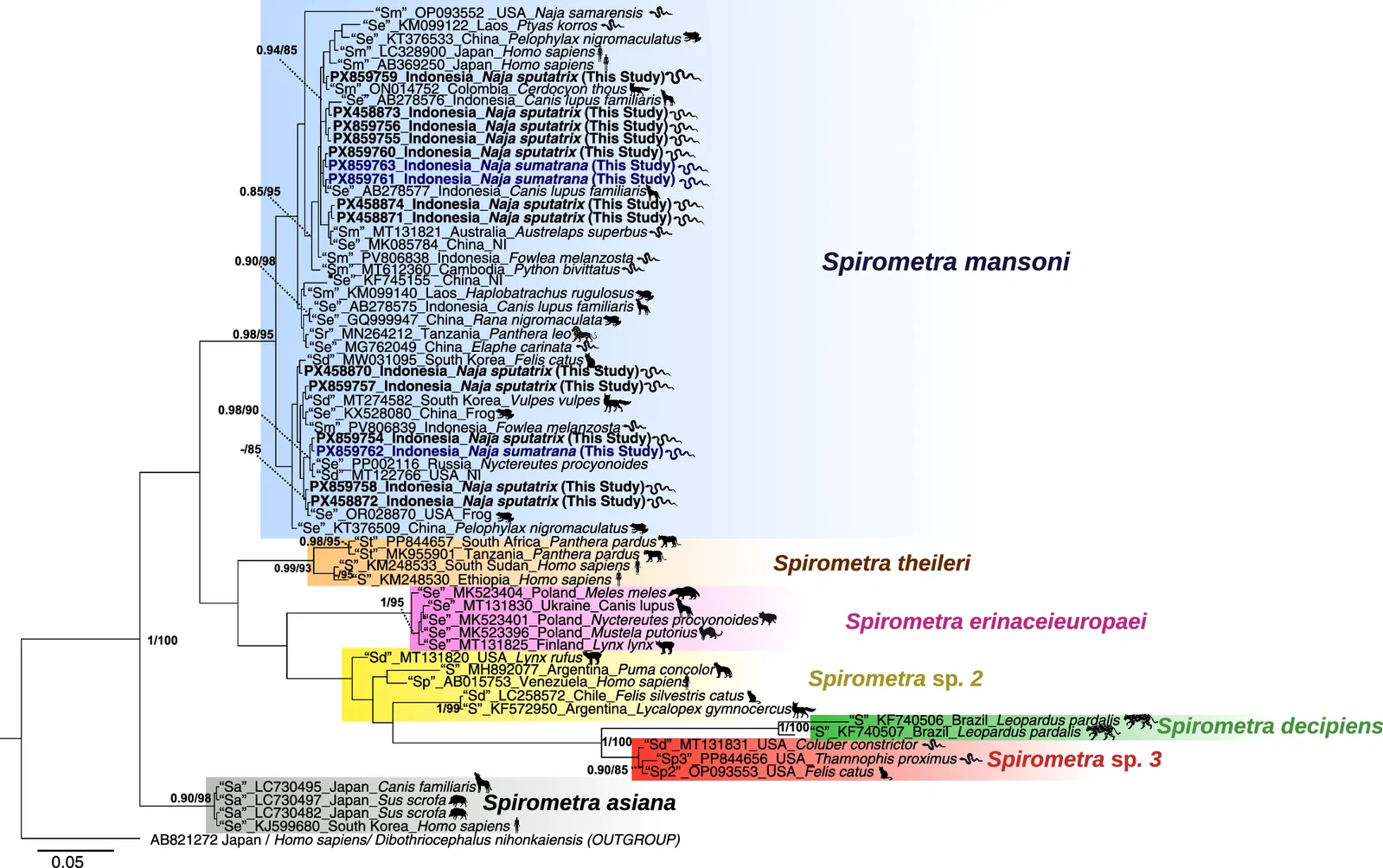
Bayesian inference (BI) phylogenetic tree inferred from partial mitochondrial cox1 sequences representing seven valid species of *Spirometra*. Scientific names that are likely misidentifications are enclosed in quotation marks and labelled using the following abbreviations: "Sm" for *S. mansoni*, "Se" for *S. erinaceieuropaei*, "Sd" for *S. decipiens*, "Sr" for *S. ranarum*, “St” for *S. theileri*, "Sa" for *S. asiana*, "Sp" for *S. proliferum*, "S" for *Spirometra* sp., "Sp3" for *Spirometra* sp. 3, and "Sp2" for *Spirometra* sp. 2. Numbers at nodes indicate posterior probabilities from the BI analysis (values ≥ 0.85 shown), followed by nodal support from Maximum Likelihood (ML) analysis (ultrafast bootstrap values ≥ 85% shown). Newly generated sequences from the present study are shown in bold. *Dibothriocephalus nihonkaiensis* (GenBank accession no. AB821272) was used as the outgroup. Scale bar indicates the expected number of substitutions per site. Details of all sequences included in the analysis are provided in Additional file 2: Table S2. NI, not indicated

Mitochondrial *cox1* sequences generated in this study (*n* = 15; ~ 440 bp per isolate; 378 aligned positions across a total dataset of 41 sequences) yielded inconsistent species assignments in BLAST searches, showing high similarity to sequences deposited under several mislabelled taxa, including *S. erinaceieuropaei*, *S. ranarum*, and *S. decipiens*. These results highlight the unreliability of similarity-based identification using public databases alone for *Spirometra* species. In contrast, BI and ML phylogenetic analyses based on *cox1* sequences unambiguously placed all newly generated isolates within the *Spirometra mansoni* clade with strong nodal support (posterior probability = 0.98; bootstrap support = 95%). Species assignments follow the revised nomenclature of Kuchta et al. [9] and Edila et al. [8], and may differ from nominal species names as deposited in GenBank, which frequently reflect outdated or erroneous identifications within this genus. The sequences formed a well-supported monophyletic group together with previously published *S. mansoni* isolates derived from a range of vertebrate hosts—including reptiles, amphibians, carnivorous mammals, and humans—collected across Asia, Australia, Africa, and the Americas (Fig. 3).

Although plerocercoids from *N. sputatrix* and *N. sumatrana* differed markedly in body size, pairwise genetic distances were consistently low across all comparisons. Within *N. sputatrix*, *P*-distances ranged from 0.000 to 0.020; within *N. sumatrana*, from 0.000 to 0.020; and between the two host species, from 0.000 to 0.020 (Table 1, Additional file 1: Table S1). The congruence of these ranges indicates no detectable host-associated genetic differentiation between the two examined cobra species. Compared to all previously published *S. mansoni* isolates from Indonesia, sequences generated in the present study showed a narrow range of genetic divergence (*P*-distance = 0.000–0.026). The maximum divergence (0.026) was observed between an isolate from *Canis lupus familiaris* from Samosir, North Sumatra (AB278576) and isolates obtained in this study from *Naja sputatrix* (PX458870, PX458872, PX859754) and *N. sumatrana* (PX859762) (Additional file 1: Table S1).

**Table 1:**
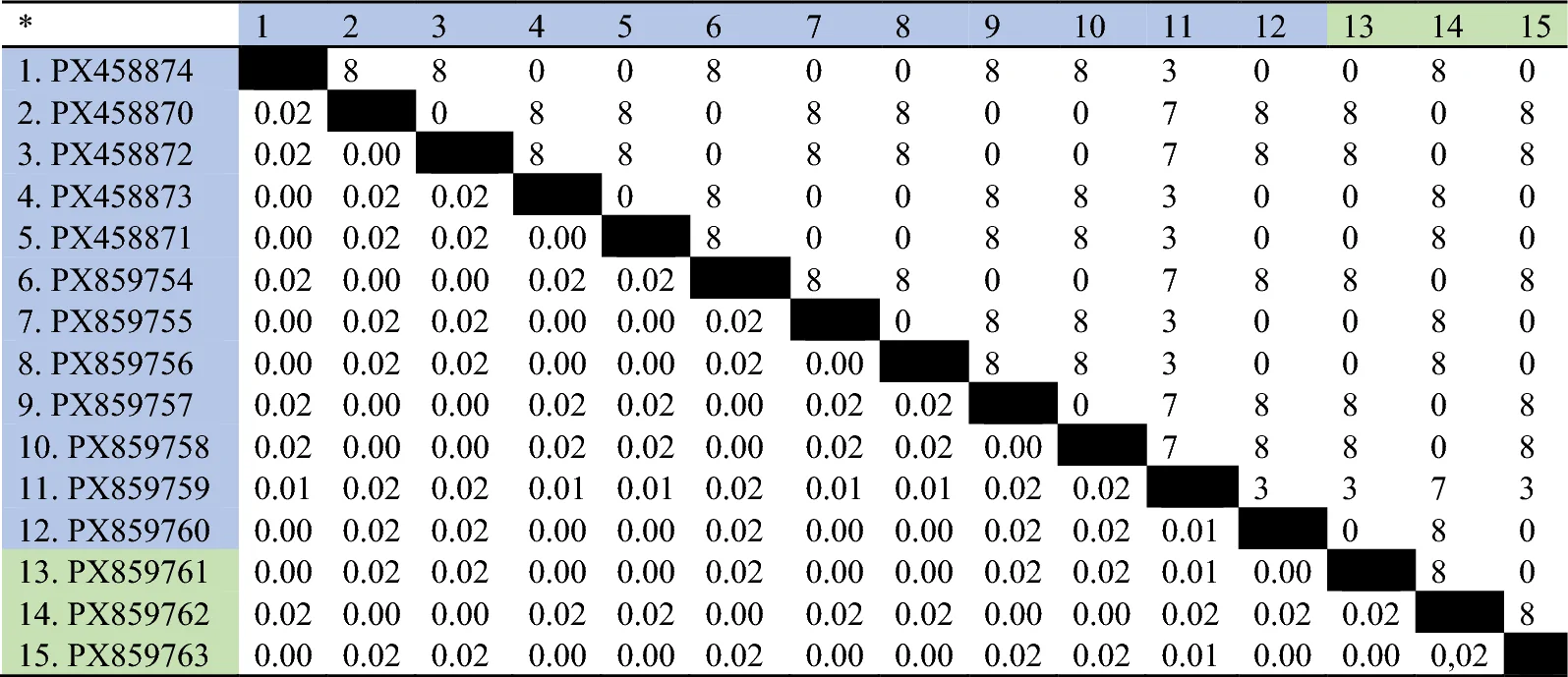
Uncorrected p-distances of partial mitochondrial *cox1* sequences among *Spirometra mansoni* isolates recovered from *Naja sputatrix* and *Naja sumatrana*, genetic distances are shown below the diagonal, whereas the number of nucleotide differences is given above the diagonal

^*^Specimens from *N. sputatrix* are highlighted in blue and those from *N. sumatrana* in green

To contextualise the newly generated sequences within the broader global diversity of *S. mansoni*, pairwise genetic distances were calculated between *Naja*-derived isolates and all previously published sequences of this species included in the dataset. At the global scale, *S. mansoni* isolates derived from *Naja* spp. in this study were genetically identical to sequences from Colombia (ON014752), Australia (MT131821), and China (MK085784). The highest divergence involving *Naja*-derived isolates was observed relative to a sequence from the United States (OP093552; *P*-distance = 0.053). Across the entire dataset, the maximum pairwise divergence detected was 0.058, occurring between an isolate from China (KF745155) and the same U.S. isolate (OP093552) (Additional file 1: Table S1).

## Discussion

This study provides the first molecular confirmation of *S. mansoni* infecting *N. sputatrix* and documents, for the first time, *Spirometra* infection in *N. sumatrana*. Macroscopically, the recovered spargana were distinguishable from other cestode larvae by the absence of a hooked scolex, suckers, or a vesicular body, and the presence of solid parenchymal tissue without a primary lacuna [11]. The cephalic region exhibited a flattened anterior end with two longitudinal bothria; however, while these features are sufficient for genus-level identification, they are insufficient for species-level diagnosis due to morphological conservatism across *Spirometra* taxa [8, 30]. Species confirmation therefore required molecular data, with cox1-based phylogenetic inference representing the current methodological standard for *Spirometra* identification [9, 26, 37]. Phylogenetic analyses based on mitochondrial *cox1* sequences consistently placed all isolates within the *S. mansoni* clade with strong nodal support, despite inconsistent species assignments retrieved from BLAST similarity searches, a finding attributable to the pervasive misidentification of *Spirometra* sequences in public databases reflecting long-standing taxonomic challenges within the genus [9, 24].

Marked differences in larval body size were observed between plerocercoids recovered from the two cobra species; however, these differences were not accompanied by genetic divergence. Pairwise p-distances were congruent across all comparisons: 0.000–0.020 within *N. sputatrix*, 0.000–0.020 within *N. sumatrana*, and 0.000–0.020 between the two host species (Table 1; Additional file 1: Table S1). Both detected cox1 haplotypes were shared across host species, providing internally consistent evidence against host-associated genetic partitioning between the two examined cobra species. The absence of host-associated differentiation indicates that larval size variation likely reflects ecological or host-related factors, such as infection duration, host physiology, or growth conditions, rather than species-level divergence [38, 39], consistent with the broad host plasticity of *S. mansoni* at the second intermediate or paratenic host level. Although reliance on a single mitochondrial marker constrains the strength of population-genetic inferences, the congruence of within- and between-host distance ranges supports this interpretation within the scope of the present dataset; multi-locus analyses would be required for broader conclusions regarding host specificity at the population level.

The genetic identity of *S. mansoni* isolates from cobras with sequences previously reported from other Indonesian vertebrates, including snakes and domestic dogs [23, 24], demonstrates that identical cox1 haplotypes circulate among multiple host taxa involved in the life cycle, suggesting shared transmission networks among these vertebrate species within the region. Isolates from *N. sputatrix* (East Java) and *N. sumatrana* (West Sumatra) exhibited p-distances of 0.000–0.020, comparable to the range observed across all Indonesian isolates (0.000–0.026), consistent with limited geographic differentiation of *S. mansoni* at the regional scale examined; formal population structure analyses would, however, be required to substantiate this inference. Recent cox1-based phylogenetic studies have clarified that the genus *Spirometra* comprises several geographically structured species-level lineages, including *S. erinaceieuropaei*, *S. decipiens*, *S. theileri*, *S. asiana*, and additional American lineages [9]. Within this framework, *S. mansoni* represents the principal Asian lineage and the most widely distributed species within the genus. The recovery of this lineage from multiple cobra species in Indonesia expands its confirmed host range and strengthens evidence of its ecological generalism among reptiles.

The close genetic similarity between Indonesian cobra-derived isolates and sequences from geographically distant regions, including Australia, China, and South America, underscores the remarkably broad distribution of *S. mansoni*. Low pairwise divergence across distant localities (*P*-distance ≤ 0.053 across the global dataset) is consistent with broad intraspecific cox1 similarity in *S. mansoni*, corroborating previous reports of its wide geographic distribution [9]. Given that analyses were based on a partial cox1 fragment (378 informative positions), conclusions regarding overall mitochondrial divergence should be interpreted with appropriate caution; as cox1 harbours the greatest mitochondrial diversity in *S. mansoni*, future studies employing the complete gene are recommended to avoid underestimation of genetic diversity.

Indonesia's position as the world's leading exporter of snakes provides a compelling anthropogenic context for understanding the broad geographic distribution of this parasite. Millions of wild-caught snakes, including cobras, are harvested annually and incorporated into domestic and international markets [3, 5, 40, 41], providing a plausible mechanism for the translocation of infected hosts across broad geographic areas. This likely contributes to the persistence of genetically similar parasite populations alongside other recognised dispersal pathways, including the movement of domestic animals [42, 43]. Critically, the very cobra species implicated in this study are among the most heavily exploited for human consumption in Indonesia [5, 7], and *S. mansoni* plerocercoids are transmitted to humans through ingestion of infected intermediate or paratenic hosts. Given the high infection prevalence documented in *N. sputatrix* across multiple surveys [8, 21] and the first confirmed infection in *N. sumatrana* reported here, cobras represent a plausible and epidemiologically relevant source of human sparganosis exposure in Indonesia and in countries receiving Indonesian snake products.

Snakes commonly harbour *Spirometra* plerocercoids without overt clinical signs, and infections in reptiles are typically detected during parasitological or molecular surveys rather than clinical investigations [11, 44]. Asymptomatic carriage increases the probability that infected individuals enter regional or international trade undetected, particularly within legal wildlife trade systems where quarantine protocols are generally designed to prevent acute infectious diseases rather than helminths transmitted through consumption of infected animal hosts [45–47]. The global herpetofauna trade, including both legal and illegal sectors, has been recognised as an important pathway for the dissemination of zoonotic pathogens [48–50].

## Conclusions

*Spirometra mansoni* was confirmed in two cobra species, *N. sputatrix* and *N. sumatrana*, representing the first molecular identification of this zoonotic cestode in both hosts in Indonesia. No host-associated genetic differentiation was detected between the two examined species, though this finding should be interpreted within the constraints of the present dataset, including limited sample size and reliance on a partial cox1 fragment that may underestimate intraspecific genetic diversity. Low cox1 divergence across Indonesian island systems suggests regional population connectivity, and large-scale wildlife exploitation and trade likely promote gene flow, facilitating the dispersal of genetically homogeneous *S. mansoni* populations across geographically distant regions. These results highlight cobras as epidemiologically relevant hosts at the interface of sylvatic transmission cycles and human exposure, and underscore the need for molecular surveillance of *Spirometra* in wildlife entering commercial trade as part of evidence-based zoonotic risk assessment and quarantine frameworks.

## Supplementary Information

Additional file 1: Table S1. Pairwise p-distance values among *Spirometra mansoni* mitochondrial cox1 sequences analysed in this study. The matrix includes all newly generated Indonesian isolates and comparative reference sequences used to assess intraspecific genetic divergence. Genetic distances were calculated as uncorrected p-distances.

Additional file 2: Table S2. Mitochondrial cox1 sequences of *Spirometra* species included in the phylogenetic analyses. Newly generated sequences from Indonesia are indicated as “This study”. Species assignments follow currently accepted nomenclature based on recent phylogenetic syntheses. NI = not indicated.

## Data Availability

The representative *cox*1 gene sequences obtained in this study were deposited in GenBank under the following accession numbers: PX458870–PX458874, PX859754–PX859760 and PX859761–PX859763.
